# Identification of the key amino acid mutations in the PB2 and PA proteins of classical swine H1N1 influenza A virus in mammalian adaptation

**DOI:** 10.1080/22221751.2025.2602310

**Published:** 2025-12-09

**Authors:** Xiuhui Wang, Xiaomin Liu, Shuaiyong Wang, Qi Wang, Juan Wang, Manzhu Wang, Yun Yao, Yanjun Zhou, Tongling Shan, Wu Tong, Hao Zheng, Ning Kong, Yifeng Jiang, Changlong Liu, Guangzhi Tong, Hai Yu

**Affiliations:** a School of Life Sciences and Food Engineering, Hebei University of Engineering, Handan, People’s Republic of China; b Shanghai Veterinary Research Institute, Chinese Academy of Agricultural Sciences, Shanghai, People’s Republic of China; c Jiangsu Co-Innovation Center for Prevention and Control of Important Animal Infectious Diseases and Zoonoses, Yangzhou, People’s Republic of China

**Keywords:** Classical swine H1N1 influenza virus, amino acid mutations, PB2-D740N, PA-T97I, mammalian adaptation

## Abstract

The classical swine (CS) H1N1 influenza virus, first isolated in 1930, is highly homologous to the 1918 Spanish influenza virus. CS H1N1 virus, which crossed the interspecies barrier to infect humans has become the dominantly prevalent strain in China’s pig population, showing a trend of continuous transmission. However, whether subsequent adaptation of CS H1N1 to mammals would increase their pathogenicity toward humans is unknown. To address this, a mouse-adapted (MA) CS H1N1 virus (A/Swine/Guangdong/1/2011[G11-MA]) was generated through serially passaged in mouse lungs, exhibiting increased virulence compared to the wild-type (WT). Further study showed that the G11-MA strain exhibited amino acid mutations in PB2-D740N, PB1-T56I, PA-T97I and HA-K188E, and the combination of PB2-D740N with PA-T97I improved the replication ability in mammalian cells and mice. The G11-MA strain with PB2 and PA (G11/MA PB2PA) group enhanced the viral polymerase activity, with a similar survival rate and weight loss of mice compared to the G11-MA group. Our study demonstrates that the combination of PB2-D740N and PA-T97I plays a key role in the virulence phenotype of CS H1N1 influenza viruses, and provides important information for evaluating the pandemic risk of swine influenza strains.

## Introduction

Influenza A virus (IAV) is a global pathogen of a wide range of mammalian and avian species, including horses, swine, canines and poultry [1]. Pigs are susceptible to avian, swine, and human IAVs, and are considered important hosts and “mixing vessels” in the generation of influenza viruses with pandemic potential [2–4]. There are three subtypes of IAVs currently circulating in pigs globally: H1N1, H1N2, and H3N2 [5,6]. China has the most complex swine influenza viruses (SIVs) ecosystem with classical swine (CS) lineage, North American triple-reassortant (TR) lineage, and Eurasian avian-like (EA) lineage [5,7,8]. CS H1N1 was first observed in 1918 in the United States, and the progenitor of this virus was the 1918 pandemic virus [9,10]. Since it was first isolated in 1930, the CS H1N1 influenza virus has been widely spread throughout the world, causing great losses to the pig industry and posing a significant challenge to public health. Since the 1970s, more than 50 human cases infected with SIV under natural conditions have been reported around the world, most caused by the CS H1N1 virus, and some even caused serious consequences [11,12]. In January 1976, a soldier infected with the CS H1N1 in New Jersey died of pneumonia, and the virus was isolated from another five soldiers very soon afterwards [13]. In 2009, studies showed that the HA, NP, and NS gene segments of the 2009 H1N1 pandemic influenza virus are of CS lineage [14,15]. Overall, the CS H1N1 virus plays a key role during the evolution of influenza viruses and frequently crosses species barriers and adapts to new hosts, indicating that research on CS H1N1 holds significant public health significance. Therefore, continuous surveillance of SIVs in pigs and an assessment of their zoonotic potential are essential for the preparedness of human pandemics [16].

Reassortment of influenza viruses is a primary way to generate progeny viruses with novel antigenic and biological characteristics, which will cause catastrophic epidemics and pandemics [16]. Several viral genetic factors contribute to the transmissibility, virulence, interspecies transmission, and pandemic potential of the IAVs. Among these, virus polymerase complex, consisting of the polymerase basic 1 (PB1), polymerase basic 2 (PB2), and polymerase acidic (PA), plays a critical role in the replication and adaptation of the viruses [17,18]. Various mutations in the polymerase complex are also associated with increased virulence, including PB2-E627K, PB2-D701N, PB2-R561K, PB2-A588V, PB2-E158G [19], A271T [20], PB1-R198K [21–23], PB1-F2-N66S [24], PA-I127V [25] and so on. Another study has shown that PA-T97I of mouse-adapted H5N2 contributes to pathogenicity in a mouse model [26]. What’s more, the PA-T97I can also enhance polymerase activity and/or increase virulence in mice either alone or in combination with other mutations in H6 subtype avian influenza viruses [27–29]. Moreover, this can also be seen in other segments of the virus, such as an H5N1 virus that derived its PA and NS genes from the 2009/H1N1 virus and is transmissible to guinea pigs [30]. Research indicates that the molecular mechanisms underlying the difference in viral biological characteristics are various.

The BALB/c mouse is a common model system used for studying the pathogenicity and immunity of influenza virus. Mice are not naturally infected with human or other strains of influenza virus, but most strains can be experimentally adapted for mouse virulence by serial lung-to-lung passages [25,30,31]. Here, we explored the impact of mammalian adaptation on the pathogenesis of CS H1N1 virus by performing 13 blind serial lung-to-lung passages in mice, and a mouse-adapted (MA) H1N1 virus (A/Swine/Guangdong/1/2011[G11-MA]) with increased virulence was obtained. Using reverse genetics, the underlying molecular determinants of MA variant were identified, indicating that PB2-D740N and PA-T97I are responsible for its higher pathogenicity *in vitro* and *in vivo*. This study demonstrates that CS H1N1 influenza virus increased the pathogenicity for mice by acquiring key mutations in PB2 and PA, and provides important insights for monitoring field strains with pandemic potential.

## Materials and methods

### Cells and viruses

Madin-Darby canine kidney (MDCK) cells, Human embryonic kidney (HEK293T) cells and human pulmonary adenocarcinoma (A549) cells were grown in Dulbecco’s modified Eagle’s medium (DMEM) with 10% fetal bovine serum and antibiotics. The CS H1N1 influenza virus A/Swine/Guangdong/1/2011(H1N1) (G11, GenBank accession no.: MT410579) was initially isolated from a sick piglet at a large-scale pig farm in Guangdong Province of China in 2011 and stored by our laboratory [32,33]. Viruses were propagated in MDCK cells at 37℃ with 5% CO_2_ for 72 h and stocked at −80℃. The 50% tissue culture infective dose (TCID_50_) of each virus was calculated by the method of Reed–Muench [34].

### Plasmid construction and reverse genetics

We used an eight-plasmid reverse genetics system for virus rescue as described previously [32]. Eight gene segments from G11-WT and four gene segments (PB2, PB1, PA, and HA mutants) from G11-MA were cloned into a pBD plasmid system and were confirmed using DNA sequencing. All recombinant and point mutation viruses (see Supplementary Figure) were rescued. Specifically, HEK293T cell monolayers in 6-well plates were transfected at 80∼90% confluency with 4 μg of the eight plasmids (500 ng of each plasmid) by using Lipofectamine 3000 (Invitrogen), according to the manufacturer’s instructions. After incubating at room temperature for 5 min, the mix of DNA and Lipofectamine 3000 was added to the cells. After 6 h, the mixture was replaced with Opti-MEM (GIBCO) containing 0.2% bovine serum albumin (BSA) and 1 μg/ml L-1-Tosylamido-2-phenylethyl chloromethyl ketone (TPCK)-treated trypsin. The supernatant was harvested and injected into 10-day-old specific-pathogen-free (SPF) embryonated eggs for virus propagation. Virus titres were determined as the TCID_50_ in MDCK cells and calculated from 3 replicates by the method of Reed–Muench [34].

### Adaptation of CS H1N1 influenza virus to mice

Three 6-week-old female BALB/c mice (Shanghai Laboratory Animal Center, China) were lightly anesthetized with diethyl ether and intranasally (i.n.) inoculated with 10^6^ TCID_50_/mL wild-type (WT) virus in a volume of 100 μL. Lungs were harvested after three days post injection and homogenized with DMEM containing penicillin and streptomycin. The homogenate was centrifuged at 10,000 rpm for 10 min at 4°C and then filtered through a 0.22-μm-pore-size cellulose acetate filter (Millipore, USA). Serial lung-to-lung passages were performed and we closely monitored the clinical symptoms and lung injury of the infected mice. Prior to the 12th passage, the mice showed no clinical or only mild symptoms, such as transient depression, a slight decrease in food intake, and then recovered and started to gain weight. Histopathological examination showed no visible changes or very mild lesions in the lung. Until the 13th passage, the mice exhibited severe clinical symptoms and ultimately died, with significant lung injury. Therefore, the lungs of the mice from the 13th passage were harvested for further studies.

### Mouse experiments

To determine the 50% mouse lethal doses (MLD_50_) value of viruses, groups of five six-week-old female BALB/c mice were i.n. inoculated with 100 μL of 10-fold serial dilutions containing 10^7^ to 10^4^ TCID_50_/mL of virus in sterile DMEM and observed for signs of morbidity over 14 days. The MLD_50_ value was calculated by the method of Reed–Muench [34]. To measure viral load in mouse lungs, groups of mice were infected with 100 μL of DMEM containing 10^6^ TCID_50_/mL of indicated viruses or 100 μL of sterile DMEM (mock group). On day 3 and 5 postinfection (p.i.), three mice from each group were euthanized and the lungs were collected for virus titration in eggs. Histology and immunohistochemistry were also performed on day 3 p.i. The remaining five mice in each group were monitored for weight loss and clinical symptoms for 14 days after infection. Mice that decreased more than 25% of their initial body weight were humanely euthanized.

### Sequence analysis

Viral RNA was extracted from the cell supernatants by the RNeasy Mini Kit (QIAGEN, Germany) according to the manufacturer’s instructions. The cDNA was synthesized and the whole genome segments were amplified by PCR using primers described previously [32]. The PCR reaction was carried out in a 50 μL volume system, including 5 μL 10×pfu Ultra II Rxn Buffer (Agilent Technologies, Beijing, China), 2 μL of each primer (10 μmol/L), 100–200 ng genomic DNA, 1 μL dNTP (Takara Bio, Dalian, China), and then made up to 50 μL with water. The PCR cycling conditions were as follows: initial denaturation at 95◦C for 5 min, followed by 40 cycles of denaturation at 95◦C for 30 s, annealing at 58◦C for 30 s, extension at 72◦C for 30 s, and a final extension at 72◦C for 15 min. The PCR products were confirmed using 1% agarose gel electrophoresis and purified using a Gel Extraction Kit (OMEGA Bio-Tek, USA). To sequence the viruses, the PCR products were cloned into the pJET 2.0 blunt-end cloning vector (Thermo Scientific USA). At least three clones per gene were sequenced by using the ABI3770 Sanger sequencing (Applied Biosystems, Foster City, CA, USA). DNA sequences were analyzed and compared to the parental G11-WT virus using the DNAStar (v17.6).

### Virus growth kinetics

To determine the difference in the growth kinetics of different viruses *in vitro*, confluent MDCK and A549 cells were infected at a multiplicity of infection (MOI) of 0.001 and 0.01, respectively. After 1 h incubation, the cells were washed twice, and then incubated with DMEM containing 0.2% BSA and TPCK-treated trypsin (1.0 μg/ml) at 37 °C with 5% CO_2_. Culture supernatants were collected at 12, 24, 36, and 48 h p.i. and viral titres were determined by the TCID_50_ assay in MDCK cells.

### Minigenome reporter assays

The polymerase activity of the influenza virus ribonucleoprotein complexes (vRNPs) was measured using the Dual-Luciferase Reporter Assay System (Promega). Concretely, the plasmids of PA, PB1, PB2, and NP (200 ng each) were mixed with a luciferase reporter plasmid (200 ng) and a thymidine kinase promoter-Renilla luciferase reporter plasmid (pRL_TK) construct (20 ng), then co-transfected into HEK293T cells in a 12-well plate with Lipofectamine 3000 (Invitrogen, Carlsbad, CA), and incubated at 37 °C for 24 h. Luciferase production was assayed using the dual-luciferase reporter assay system (Promega) to measure firefly and Renilla luciferase activities according to the manufacturer’s instructions. All results are shown as the means ± standard deviation (SD) of the representative results from three independent experiments and are standardized to the parental rG11-WT (100%).

### Polymerase stability analysis

To assess the impact of amino acid mutation on the stability of the viral polymerase, we used ChimeraX v1.10 to evaluate the formation and length of hydrogen bonds. Specifically, we first obtained the influenza virus polymerase with the complete sequences from the Protein Data Bank website (https://www.rcsb.org/), and then imported it into Chimera X, selecting amino acids within a 5 Angstroms (Å) range of the mutation site, and simulating the number and length of hydrogen bonds before and after the mutation.

### Ethics statement and facility

The present study was carried out in strict accordance with the recommendations in the Guide for the Care and Use of Laboratory Animals of the Ministry of Science and Technology of the People’s Republic of China. All studies with live viruses were carried out in a biosecurity level 2 laboratory approved for such use by the Shanghai Veterinary Research Institute (SVRI) of the Chinese Academy of Agricultural Sciences (CAAS). All animal experiments were reviewed and approved by the SVRI and were carried out in accordance with the guidelines of SVRI, Shanghai, China (permit number SYXK 2016-0117). The protocol was approved by the Committee on the Ethics of Animal Experiments of the SVRI of the CAAS.

## Results

### Adaptation of CS H1N1 influenza virus in mice

The CS H1N1 virus is almost avirulent in mice after the initial infection. To investigate the adaptation of the virus to mice, we produced MA variants of the A/Swine/Guangdong/01/2011 (H1N1) strain by serial lung-to-lung passages in mice. After 13 passages, all inoculated mice showed severe clinical symptoms including dyspnoea, huddling, ruffled fur, hunched posture, and drastic weight loss; the lung tissue lesions that exhibited extensive bleeding, hyperaemia and parenchyma died within 3 days. These results indicated that the MA variants of CS H1N1 isolates had acquired mutations that profoundly affect virulence. The virus of the 13th passage presented in the lung homogenate, designated as G11-MA, was purified three times by using limited dilution assays (LDA) in MDCK cells.

**Figure 1.**
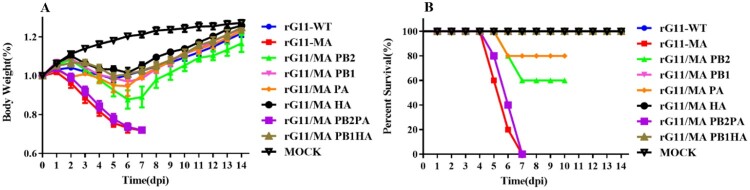
The pathogenicity of the mutants in mice. Groups of eleven 6-week-old female BALB/c mice were lightly anesthetized with diethyl ether lightly and i.n. inoculated with 100 μL of DMEM containing 10^6^ TCID_50_/mL of indicated viruses or 100 μL of sterile DMEM (mock group) to monitored for weight loss and clinical symptoms for 14 days after infection. Mice losing more than 25% of their initial body weight were humanely euthanized. (A) Weight change in mice. (B) Survival curve of mice.

To compare the virulence of rG11-MA and rG11-WT, 6-week-old female BALB/c mice were i.n. inoculated with the indicated viruses. The MLD50 of rG11-WT and rG11-MA were 6.5 and 3.6, respectively (Table 1). To further clarify the pathogenicity of each strain, groups of five mice were i.n. inoculated with 10^6^ TCID_50_/ml of virus. Survival rate and body weight were measured daily for two weeks. No morbidity or mortality was observed in mice infected with rG11-WT. However, mice infected with the rG11-MA exhibited clinical symptoms of disease, including huddling, hunched posture, ruffled fur, and lost weight progressively, all died within 7 days (Figure 1). These results showed that rG11-MA was significantly more virulent than rG11-WT and lethal to mice.

**Table 1.** Virulence of various viruses in mice.

Virus	Mortality (%)	MLD_50_ (log_10_ TCID_50_)
G11-WT	0	>6.5
G11-MA	100%	3.3
rG11-WT	0	>6.5
rG11-MA	100%	3.6
rG11/MA PB2	40%	5.3
rG11/MA PB1	0	>6.5
rG11/MA PA	20%	5.7
rG11/MA HA	0	>6.5
rG11/MA PB2 PB1	60%	4.8
rG11/MA PB2 PA	100%	3.8
rG11/MA PB2 HA	40%	5.2
rG11/MA PB1 PA	20%	5.4
rG11/MA PB1 HA	0	>6.4
rG11/MA PA HA	20%	5.5
rMA/G11 PB2	40%	5.2
rMA/G11 PB1	100%	3.8
rMA/G11 PA	80%	4.6
rMA/G11 HA	100%	3.6

### Four amino acids contribute to the difference in the replication and pathogenicity of rG11-WT and rG11-MA

In order to further investigate the host adaptation and pathogenic mechanism of classical H1N1 SIV to mice, the sequences and amino acid differentiation of G11-WT and G11-MA were analyzed. The results showed that the G11-MA had mutations at four amino acid sites, namely PB2 (D740N), PB1 (T56I), PA (T97I), and HA (K188E). To identify the gene(s) contribute to the pathogenicity of G11-MA, we then generated ten single- or double- gene reassortants between G11-WT and G11-MA, and evaluated their virulence *in vitro* and *in vivo*. These reassortants were designated as rG11/MA PB2 (rG11 is the backbone and the PB2 is from MA), rG11/MA PB1, rG11/MA PA, rG11/MA HA, rG11/MA PB2PB1, rG11/MA PB2 PA, rG11/MA PB2 HA, rG11/MA PB1 PA, rG11/MA PB1 HA, and rG11/MA PA HA, respectively. Meanwhile, four reassortants were using G11-MA as the backbone and designated as rMA/G11 PB2 (rMA is the backbone and the PB2 is from G11), rMA/G11 PB1, rMA/G11 PA, and rMA/G11 HA (Supplementary figure). MLD_50_ values and the pathogenicity in mice were assessed to evaluate the biological characteristics of these recombinants (Table 1). We found that all mice inoculated with rG11-MA PB2 PA were died, and the MLD_50_ value of the virus was 3.8 log_10_ TCID_50_, which was close to that of rG11-MA (MLD_50_: 3.6 log_10_ TCID_50_). Viruses containing one of the G11-WT-associated segments in a G11-MA background (rMA/G11 PB1 and rMA/G11 HA) were the same as that of rG11/MA PB2 PA and rG11-MA, respectively. The pathogenicity of rG11-MA PB2 PA was increased almost 1,000-fold compared with rG11-WT in mice (MLD_50_: 3.8 log_10_ TCID_50_ versus 6.5 log_10_ TCID_50_), whereas the MLD_50_ values of the other nine single-gene reassortants, which ranged from 4.8 log_10_ TCID_50_ to 6.5 log_10_ TCID_50_ (Table 1)_._

### PB2-D740N and PA-T97I collectively contribute to the pathogenicity of G11-WT and G11-MA in mice

To determine the replicate ability between the rG11-WT and the mutants, groups of five BALB/c mice were i.n. inoculated with 10^5^ TCID_50_ of the virus and were monitored daily for 14 days (Figure 1). All the mice infected with rG11-MA and rG11/MA PB2 PA progressively lost weight, exhibited clinical symptoms of disease (including decreased activity, huddling, hunched posture, and ruffled fur), and all died within 7 d p.i.. In contrast, no mortality was observed in mice infected with rG11-WT, rG11/MA PB1, rG11/MA HA, and rG11/MA PB1 HA, the maximum weight loss occurred at 6 d p.i., and the weight gradually increased over the remainder of the observation period (Figure 1(A)). The survival rates of rG11/ MA PB2 and rG11/MA PA groups were 60% and 80%, respectively (Figure 1(B)). This suggests that both PB2-D740N and PA-T97I could enhance the virulence of the virus, and PB2-D740N is more severe than PA-T97I. The combination of PB2-D740N and PA-T97I is responsible for the higher virulence of rG11-MA in mice. Viruses were isolated from mice and the whole genomes of viruses were sequenced, demonstrating that no other mutations were detected.

To determine whether the increased pathogenicity was correlated with the level of virus replication in mice, the virus titres in lung of mice i.n. inoculated with different viruses were detected. The viral titre of rG11-MA was significantly higher than that of rG11-WT at both 3 d (3.5 vs 5.5 log_10_TCID_50_/ml) and 5 d (3.5 vs 5.9 log_10_TCID_50_/ml) p.i. At the same time, we found that the viral titres of the viruses, except for rG11/MA PA group on 3 d p.i., containing MA PB2 and/or PA, were no less than 3.9 log_10_TCID_50_/ml on 3 d p.i. and 4.0 log_10_TCID_50_/ml on day 5 d p.i. In contrast, the virus titre of the strains with neither of these two segments were did not exceed 3.9 log_10_TCID_50_/ml on day 3 nor 4 log_10_TCID_50_/ml on day 5. Some strains, such as rMA/G11 PA, rG11-MA, rG11/MA PB2 PA, rMA/G11 PB1 and rMA/G11 HA replicated in the lungs was similar to the rG11-MA and the virus titre was 4.9, 5.5, 5.4, 5.3, and 5.7 at 3 d p.i., respectively, reaching mean peak titres≥10^4.9^ TCID_50_/ml at 3 d p.i. challenged with a dose of 10^6^ TCID_50_. Meanwhile, the viral titre of these five viruses at 5 d p.i. is 5.1, 5.9, 5.8, 5.8, and 6.0, respectively, and reaching mean peak titres≥10^5.8^ TCID_50_/ml, which is significantly higher than that of rG11-WT (*p* < 0.001). The viral titre of each strain are shown in Table 2. These results showed that the combination of PB2-D740N with PA-T97I in the rG11 backbone led to a more virulent variant in the lung of mice.

**Table 2.** Replication ability of the various mutants in the lungs of mice on days 3 and 5 p.i. All results are shown as the means ± standard deviation (SD) of the representative results from three independent experiments.

Virus	Mean virus titre (log_10_ TCID_50_/ml) ± SD	
	3 d p.i.	5 d p.i.
rG11-WT	3.5 ± 0.1	3.5 ± 0.1
rG11-MA	5.5 ± 0.3	5.9 ± 0.1
rG11/MA PB2	4.0 ± 0.1	4.3 ± 0.1
rG11/MA PB1	3.8 ± 0.1	3.9 ± 0.0
rG11/MA PA	3.7 ± 0.1	4.1 ± 0.3
rG11/MA HA	3.5 ± 0.0	3.5 ± 0.1
rG11/MA PB2 PB1	4.3 ± 0.1	4.6 ± 0.1
rG11/MA PB2 PA	5.4 ± 0.1	5.8 ± 0.2
rG11/MA PB2 HA	4.2 ± 0.0	4.4 ± 0.1
rG11/MA PB1 PA	4.1 ± 0.2	4.5 ± 0.3
rG11/MA PB1 HA	3.7 ± 0.0	3.8 ± 0.3
rG11/MA PA HA	3.9 ± 0.1	4.0 ± 0.1
rMA/G11 PB2	4.6 ± 0.4	4.8 ± 0.1
rMA/G11 PB1	5.3 ± 0.1	5.8 ± 0.3
rMA/G11 PA	4.9 ± 0.1	5.1 ± 0.3
rMA/G11 HA	5.7 ± 0.1	6.0 ± 0.1

**Figure 2.**
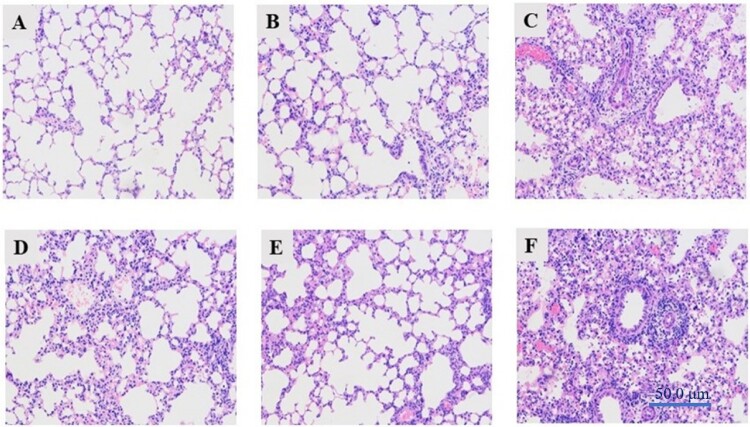
Histopathology of the lungs of inoculated mice on days 3 p.i.. Groups of three 6-week-old female BALB/c mice were lightly anesthetized with diethyl ether lightly and i.n. inoculated with 100 μL of DMEM containing 10^6^ TCID_50_/mL of indicated viruses to test the histopathology of the lungs. (A) Mock. (B) rG11-WT. (C) rG11-MA. (D) rG11/MA PB2. (E) rG11/MA PA. (F) rG11/MA PB2PA. Magnification, ×400.

To further evaluate the pathogenicity of PB2-D740N and PA-T97I amino acid mutation to CS H1N1, histopathology of lungs of inoculated mice was conducted at 3 d p.i. (Figure 2). The rG11-WT group appeared almost normal (Figure 2(B)); however, mice inoculated with rG11/MA PB2, rG11/MA PA showed inflammatory cell infiltration and moderate thickening of the alveolar walls (indicated by the arrow) (Figure 2(D,E)). The mice inoculated with rG11-MA and rG11/MA PB2 PA showed more serious lung tissue damage, with haemorrhage, lymphocyte infiltration, severe peribronchiolar inflammation, and localized interstitial pneumonia compared with rG11/MA PB2 and rG11/MA PA (Figure 2(C,F)). The histopathology and immunohistochemistry analysis results suggested that the substitutions in the PB2 and PA proteins play critical roles in inflammation in the lungs, and the combination of PB2 and PA accelerated viral replication in alveolar epithelial cells at 3 d p.i.

### The PB2-D740N and PA-T97I mutations increase G11 viral replication in mammalian cells

**Figure 3.**
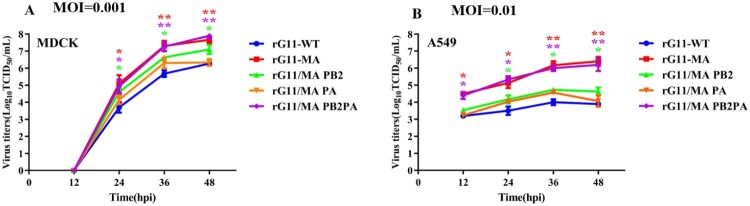
Growth kinetics of the mutants on MDCK and A549 cells. Confluent MDCK (A) and A549 (B) cells were infected at a multiplicity of infection (MOI) of 0.001 and 0.01, respectively. After 1 h incubation, the cells were washed twice, and then incubated with DMEM containing 0.2% BSA and TPCK-treated trypsin (1.0 μg/ml) at 37 °C with 5% CO_2_. Culture supernatants were collected at 12, 24, 36 and 48 h p.i. and viral titres were determined by the 50% tissue culture infective dose (TCID_50_) assay in MDCK cells. Values are shown as the means ± standard deviation (SD) of the representative results from three independent experiments and are standardized to the mock of rG11-WT. The green, purple, and red asterisks represent the viral titre differences of rG11/MA PB2, rG11/MA PB2 PA, and rG11-MA compared to rG11-WT at different time points, respectively. Differences between experimental groups were determined by using an unpaired t-test (*p < 0.05; **p < 0.01; ***p < 0.001) and analysis of variance (ANOVA) in the GraphPad Prism version 8.0 (GraphPad Software Inc. CA, USA).

To investigate the characterization of the G11-MA *in vitro*, growth kinetics were performed on MDCK and A549. The rG11-MA and rG11/MA PB2 PA replicated more efficiently in these two cells, as compared with rG11-WT, showing a significantly higher titre at 24, 36, and 48 h p.i. (Figure 3). rG11/MA PB2 and rG11/MA PA slightly increased virus replication when compared to rG11/MA, but neither could achieve the effect of the rMA/G11 PB2 PA virus. These results showed that the rG11/MA PB2 PA virus and the parental rG11-MA virus had similar replication abilities in both A549 and MDCK cells, and the combination of PB2 -D740N with PA-T97I can enhance the replication ability of the viruses in mammalian cells.

### The combination of the PB2-D740N and PA-T97I significantly increases the polymerase activity of G11-WT

**Figure 4.**
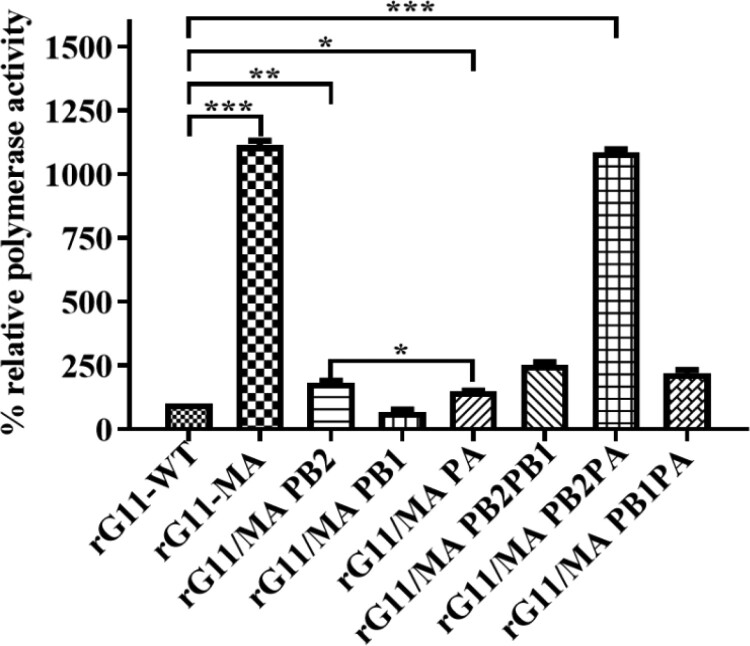
Polymerase activity of the influenza virus ribonucleoprotein complexes (vRNPs). The plasmids of PA, PB1, PB2 and NP (200 ng each) were mixed with a luciferase reporter plasmid (200 ng) and a thymidine kinase promoter-Renilla luciferase reporter plasmid (pRL_TK) construct (20 ng), then co-transfected into HEK293 T cells in a 12-well plate with Lipofectamine 3000 (Invitrogen, Carlsbad, CA), and incubated at 37°C for 24 h. Luciferase production was assayed using the dual-luciferase reporter assay system (Promega) to measure firefly and Renilla luciferase activities according to the manufacturer’s instructions. All results are shown as the means ± standard deviation (SD) of the representative results from three independent experiments and are standardized to the parental rG11-WT (100%). Differences between experimental groups were determined by using an unpaired *t*-test (*p < 0.05; **p < 0.01; ***p < 0.001) and analysis of variance (ANOVA) in the GraphPad Prism version 8.0 (GraphPad Software Inc. CA, USA).

To investigate whether the difference in pathogenicity between G11-WT and G11-MA is associated with the polymerase activity of their vRNP complex, we compared the polymerase activity using a luciferase minigenome assay in HEK293 T cells. We found that the polymerase activity of the vRNP complex of rG11/MA PB2 and rG11/MA PA was 2.2-fold and 2.1-fold greater than that of rG11-WT, respectively. In contrast, the polymerase activity of rG11/MA PB1 was reduced to 80%. Then the two segments of the vRNP complex of G11-WT with G11-MA were replaced, showing that the polymerase activities of G11-PB1PA and G11-PB2PB1 were 2.4-fold and 2.6-fold greater than those of rG11-WT, respectively. Moreover, the polymerase activity of G11-PB2PA was 11.2-fold–almost identical to that of G11-MA – and statistically significantly different (*p* < 0.001). The polymerase activity of rG11/MA PB2 was 1.23-fold greater than that of rG11/MA PA (*p* < 0.05), indicating that there is a certain difference in polymerase activity between the two mutations (Figure 4). Taken together, our results indicate that PB2-D740N and PA-T97I contributes to the polymerase activity of the G11-WT virus.

### The PB2-D740N mutation makes the polymerase more stable

**Figure 5.**
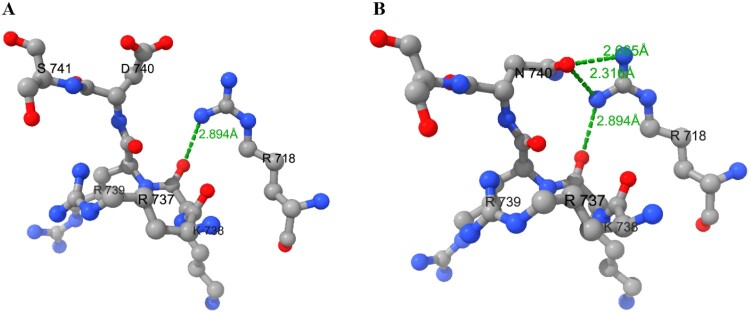
Hydrogen bond analysis of PB2-D740N. ChimeraX v1.10 software were used to analyze the change of hydrogen bonds within the 5 Angstroms (Å) range of PB2-D740N mutation. (A) The hydrogen bonds within 5 Å of PB2-D740 (PDB: 6EVK) and (B) PB2-740N, respectively. Green dot line indicates the hydrogen bond.

Analysis of the influenza virus polymerase (PDB ID 6EVK) using ChimeraX revealed that the hydrogen bonds were changed within the 5 Å range of PB2-D740N mutation (Figure 5). To PB2-D740, only one hydrogen bond was formed, that is between the R718 and the K738, forming an N–H**^ … ^**O hydrogen bond with a length of 2.894 Å (Figure 5(A)); However, PB2-740N has three hydrogen bonds within the 5 Å range. In addition to retaining the hydrogen bond from the unmutated state, 740N forms two additional hydrogen bonds with the NH1 and NH2 groups of R718, and the lengths were 2.316 Å and 2.665 Å, respectively (Figure 5(B)). The result indicates that the polymerase structure with PB2-740N is more stable compared to PB2-D740. Therefore, we infer that there is a trend of PB2-D740 to PB2-740N. In our study, even without the synergistic effect of PA-T97I, the pathogenicity of influenza virus with PB2-D740N is significant both *in vitro* and *in vivo*. Therefore, monitoring and response measures should be implemented for mutations at this site. Therefore, we recommend focusing on the PB2-D740N mutation and, implementing monitoring and prevention measures.

## Discussion

The pathogenicity and the host range of infection of IV are influenced by a variety of factors that have not yet been fully revealed. IAVs increase their host range to infect new hosts via a high mutation rate, reassortment of genome segments and antigenic shifts [31]. Mouse adaptation has been confirmed to be an effective way to find amino acid changes that affect the virulence and replication ability of influenza viruses [35–37]. However, whether or not the CS H1N1 influenza virus could become pathogenic in mice and other mammals was not known. In this study, after 13 blind serial lung-to-lung passages of CS H1N1 influenza virus in mice, a virulent MA virus (G11-MA) was obtained. This virus differed from G11 by 4 amino acid substitutions in 4 gene segments (PB2-D740N, PB1-T56I, PA-T97I, and HA-K188E). Previous studies found that PA-T97I plays an important role in the pathogenicity of AIV during its adaptation to new mammalian hosts [36]. Here, we found that the impact of the virus with the single mutation of PA-T97I is less than the virus with only PB2-D740N. The virus with the combination of substitutions of PB2-D740N and PA-T97I replicated much better with higher titres than G11 in the lungs of infected mice. These results indicated that a new determinant of pathogenicity for CS H1N1 viruses significantly enhances viral pathogenicity in mice. This phenomenon is consistent with other studies that found that the interaction of multiple sites in the polymerase complex genes is an important molecular mechanism for viral virulence, and the emergence of this combination in our study makes the host adaptation and pathogenesis of influenza virus to mammals more complex.

Influenza A virus RNA polymerase complex assembles into a central polymerase core and several flexible domains. The former is composed of PB1, the C-terminal of PA (PA-C) and the N-terminal of PB2 subunit (PB2-N), and the latter is composed of the N-terminal endonuclease domain of PA and the C-terminal two-thirds of PB2 (PB2-C). Research has showed that the flexible region around the core is related to the function of the polymerase and some sites have already been detected [38,39].

PA is involved in the endonuclease activity and PA-T97 is located in the endonuclease region (residues 1-177) of PA-N [17,40]. The endonuclease activity is of vital importance to capture capped primers from host mRNA to start mRNA transcription [38]. Meanwhile, PA-T97 located in the binding region of PA and PB2 protein, and therefore may also affect the interaction between these two proteins [41].

The 627 domain (residues 538-677) and the linear nuclear localization signal (NLS) (residues 689-759) domain form the C-terminus of PB2, and these two domains forming a tightly packed heterodimer to bind importin α [42]. PB2 is imported into the host cell nucleus independently before polymerase reconstitution by NLS. PB2-D740 is located in NLS and may affect PB2 entry into the cell nucleus, and further affect the life activities of the virus. Hydrogen bonds are one of the essential conditions for the formation of protein spatial structures. Pauling et al. [43,44] had been proposed that the hydrogen bond may be the key force in protein folding and stability. Research also indicated that the number of hydrogen bonds between residues is one of the most important analyses to evaluate the stability of a protein [45]. In our study, the number of hydrogen bonds increased with the mutation of PB2-D740N, suggesting that PB2-D740N affects the stability of the polymerase and consequently affect the pathogenicity of the virus.

PA-T97I and PB2-D740N belong to the flexible domain and have obvious synergistic effects in this study, we speculated that PB2-D740N was used to improve the nucleation efficiency of the virus protein through PA-T97I mediated interaction between PA and the PB2 protein, thereby improving the polymerase activity of the virus, promoting the transcription and replication of the virus, and thus improving the adaptability and pathogenicity of the virus to mice.

Previous study had shown that the PA-T97I was a potential virulence marker with direct evidence of contact transmission of H6 avian influenza viruses [27]. In this study, both PA-T97I and PB2-D740N were detected in CS H1N1 influenza virus for the first time and, the combination of PB2-D740N and PA-T97I has not been reported before. Therefore, circulating in pigs for a period, the CS H1N1 strain is likely to mutate into a highly pathogenic strain. Consequently, regular monitoring of CS H1N1 influenza is essential to prevent the occurrence of a pandemic. However, the transmissibility of G11-MA remains unknown, whether it leads to enhance the transmissibility and subsequently trigger a CS H1N1 influenza pandemic needs further study.

## Supplementary Material

Supplementary Figure_clean.docx
