# Supplementary material for: Identification of the key amino acid mutations in the PB2 and PA proteins of classical swine H1N1 influenza A virus in mammalian adaptation
Source: Emerg Microbes Infect. 2025 Dec 9;15(1):2602310. doi: 10.1080/22221751.2025.2602310 (PMC12777772; doi:10.1080/22221751.2025.2602310)
Supplement: Supplementary Figure_clean.docx [file TEMI_A_2602310_SM8889.docx]

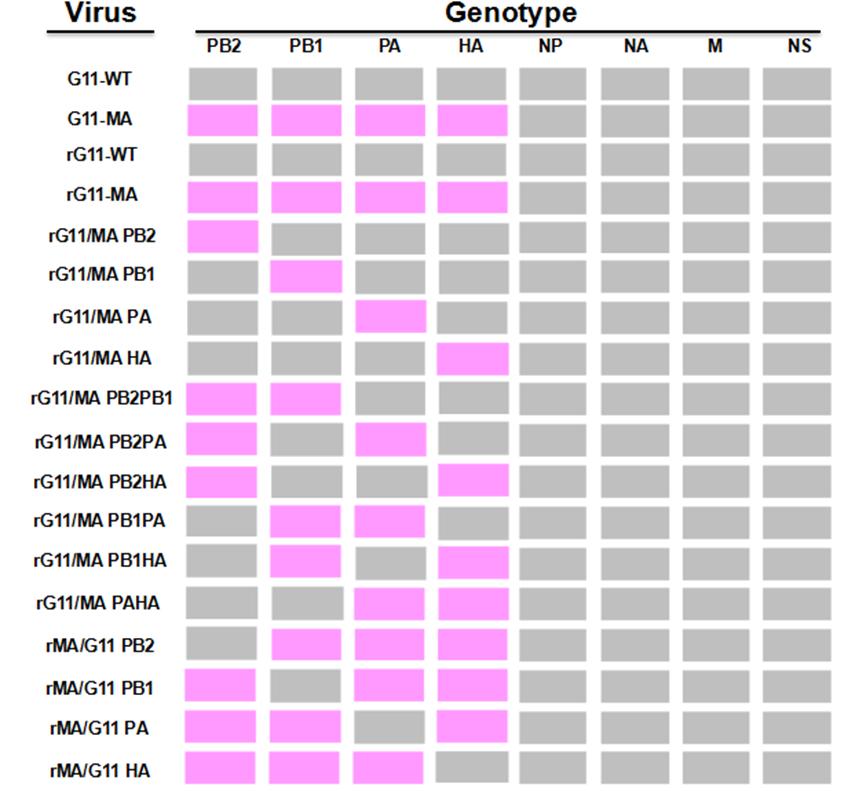


Supplementary Figure. The diagram of recombinant viruses rescued by reverse genetics system. Eight gene segments from G11-MA were cloned into a pBD plasmid system and were confirmed using DNA sequencing. The PB2, PB1, PA and HA mutants were amplified by RT-PCR and sequenced. HEK293T cells monolayers in 6-well plates were transfected at 80~90% confluency with 4 μg of the eight plasmids (500 ng of each plasmid) by using Lipofectamine 3000 (Invitrogen) according to the manufacturer’s instructions. After incubated at room temperature for 5 min, the mix of DNA and Lipofectamine 3000 were added to the cells. After 6 h later, the mixture was replaced with Opti-MEM (GIBCO) containing 0.2% bovine serum albumin (BSA) and 1 μg/ml TPCK-treated trypsin. The supernatant was harvested and injected into SPF embryonated eggs after 48 h later for virus propagation. Viruses were titrated in embryonated eggs using hemagglutination assays and sequenced.

Notes: (1)G11-WT: The wild-type (WT) of the classical (CS) H1N1 influenza virus (A/Swine/Guangdong/1/2011[H1N1, G11]) used in this study.

(2)rG11-WT: The rescue strain of G11-WT obtained through reverse genetic system, and the sequences of the eight gene segments are identical to that of G11-WT.

(3)G11-MA: The mouse-adapted (MA) CS H1N1 virus A/Swine/Guangdong/1/2011, which was generated from G11-WT by 13 blind serial lung-to-lung passages in mice. Compared to G11-WT, there are four gene mutations: PB2-D740N, PB1-T56I, PA-T97I, and HA-K188E.

(4)rG11-MA: The rescue strain of G11-MA obtained through reverse genetic system, and the sequences of the eight gene segments are identical to that of G11-MA.

(5)rG11/MA PB2: rG11 is the backbone and the PB2 is from MA.

(6)rG11/MA PB2PB1: rG11 is the backbone, and the PB2 and PB1 are from MA.

(7)rMA/G11 PB2: rMA is the backbone and the PB2 is from G11.
